# Differential expression of PD-1 and Tim-3 marks activation versus exhaustion status of T cells in the tumor microenvironment

**DOI:** 10.1186/2051-1426-2-S3-P220

**Published:** 2014-11-06

**Authors:** Jing Li, Robert L Ferris

**Affiliations:** 1 Department of Pharmacy, School of Medicine, Tsinghua University, Beijing, China, Pittsburgh, PA, USA; 2 University of Pittsburgh Cancer Institute, Pittsburgh, PA, USA

## 

Programmed Death 1 (PD-1) and T cell Ig and mucin domain-3 protein (Tim-3) are two immune checkpoint receptors (ICR) highly co-expressed on tumor infiltrating T lymphocytes (TIL). PD-1 has been shown to inhibit T cell activation and type 1 T cell responses, while Tim-3 has been proposed as a further marker of exhaustion on TIL [1,2], leading us to investigate the phenotypic and functional characteristics of TIL with differential PD-1 and Tim-3 expression from head and neck cancer (HNC) patients. Our data showed that PD-1^+^Tim-3^+ ^CD8^+ ^and Foxp3^- ^CD4^+ ^TILs manifested high phosphorylated signal transducers and activators of transcription 1 (p-STAT1) and the associated Th1 transcription factor T-bet, which might correlate with T cell exhaustion, both at baseline and upon TCR stimulation. Moreover, the sorted PD-1^+^Tim-3^+ ^CD8^+ ^TILs expressed the lowest IFN-γ and TNF-α transcripts and the least amount of secreted IFN-γ upon TCR stimulation, indicating they are the most dysfunctional T cells in the tumor microenvironment (TME). Among CD4^+^CD25^lo/- ^TIL subsets, PD-1^hi^Tim-3^- ^cells are more defective in terms of IFN-γ expression. Sorted PD-1^int^Tim-3^- ^CD8^+ ^and CD4^+^CD25^lo/- ^TILs showed higher TCR-stimulated expression of IFN-γ and TNF-α transcripts and secretion of IFN-γ, suggesting they are the most activated subsets. In addition, sorted PD-1^+^Tim-3^+ ^and PD-1^hi^Tim-3^- ^TIL were less proliferative than other subsets, concomitant with lower expression of phosphorylated S6 (p-S6), while PD-1^int^Tim-3^-^, PD-1^-^Tim-3^+ ^and PD-1^-^Tim-3^- ^TIL retained p-S6 activation or proliferation, suggesting that high expression of PD-1 on T cells interferes with TCR or Tim-3 signaling and associated cellular activation status. Taken together, PD-1^+^Tim-3^+ ^and PD-1^hi^Tim-3^- ^TIL are most dysfunctional, while PD-1^int^Tim-3^- ^TIL are more activated in terms of both Th1 cytokine production and proliferation. These results provide a better understanding of the functional status of TIL subsets and roles of PD-1 and Tim-3 in regulating anti-tumor T cell response, as targets for cancer immunotherapy.
